# Chatbot-Delivered COVID-19 Vaccine Communication Message Preferences of Young Adults and Public Health Workers in Urban American Communities: Qualitative Study

**DOI:** 10.2196/38418

**Published:** 2022-07-06

**Authors:** Rose Weeks, Lyra Cooper, Pooja Sangha, João Sedoc, Sydney White, Assaf Toledo, Shai Gretz, Dan Lahav, Nina Martin, Alexandra Michel, Jae Hyoung Lee, Noam Slonim, Naor Bar-Zeev

**Affiliations:** 1 International Vaccine Access Center Department of International Health Johns Hopkins Bloomberg School of Public Health Baltimore, MD United States; 2 Center for American Indian Health Department of International Health Johns Hopkins Bloomberg School of Public Health Baltimore, MD United States; 3 Stern School of Business New York University New York, NY United States; 4 Whiting School of Engineering Johns Hopkins University Baltimore, MD United States; 5 IBM Research AI Haifa Israel; 6 Department of International Health Johns Hopkins Bloomberg School of Public Health Baltimore, MD United States

**Keywords:** vaccine hesitancy, COVID-19, chatbots, AI, artificial intelligence, natural language processing, social media, vaccine communication, digital health, misinformation, infodemic, infodemiology, conversational agent, public health, user need, vaccination, health communication, online health information

## Abstract

**Background:**

Automated conversational agents, or chatbots, have a role in reinforcing evidence-based guidance delivered through other media and offer an accessible, individually tailored channel for public engagement. In early-to-mid 2021, young adults and minority populations disproportionately affected by COVID-19 in the United States were more likely to be hesitant toward COVID-19 vaccines, citing concerns regarding vaccine safety and effectiveness. Successful chatbot communication requires purposive understanding of user needs.

**Objective:**

We aimed to review the acceptability of messages to be delivered by a chatbot named VIRA from Johns Hopkins University. The study investigated which message styles were preferred by young, urban-dwelling Americans as well as public health workers, since we anticipated that the chatbot would be used by the latter as a job aid.

**Methods:**

We conducted 4 web-based focus groups with 20 racially and ethnically diverse young adults aged 18-28 years and public health workers aged 25-61 years living in or near eastern-US cities. We tested 6 message styles, asking participants to select a preferred response style for a chatbot answering common questions about COVID-19 vaccines. We transcribed, coded, and categorized emerging themes within the discussions of message content, style, and framing.

**Results:**

Participants preferred messages that began with an empathetic reflection of a user concern and concluded with a straightforward, fact-supported response. Most participants disapproved of moralistic or reasoning-based appeals to get vaccinated, although public health workers felt that such strong statements appealing to communal responsibility were warranted. Responses tested with humor and testimonials did not appeal to the participants.

**Conclusions:**

To foster credibility, chatbots targeting young people with vaccine-related messaging should aim to build rapport with users by deploying empathic, reflective statements, followed by direct and comprehensive responses to user queries. Further studies are needed to inform the appropriate use of user-customized testimonials and humor in the context of chatbot communication.

## Introduction

Vaccine hesitancy has emerged as a public health threat as trust in immunization systems has been strained across much of the world [1,2]. Global measles outbreaks occurring in the face of waning vaccine uptake propelled vaccine hesitancy onto the World Health Organization’s list of top global health concerns [3,4]. The COVID-19 pandemic has brought hesitancy into sharp focus, including in the United States, which experienced one of the highest COVID-19 mortality rates among high-income nations [5].

A survey of more than 5 million Americans conducted via Facebook found that adults aged 18-34 years had the highest rates of vaccine hesitancy in May 2021 [6]. Moreover, despite disproportionately high COVID-19 mortality rates within communities of color [7], younger adults and Black, American Indian or Alaska Native, and multiracial groups continued to be the most hesitant, citing concerns regarding vaccine development, safety, and effectiveness [8-11]. As of May 2022, 3 in 10 Americans eligible for a COVID-19 vaccine have yet to get fully vaccinated, fueling continued disease spread and hindering pandemic recovery efforts [12].

To combat hesitancy, agency and advocacy leaders drew upon decades of communication science learning about building vaccine demand. Such guidance included the need for proactive planning efforts to understand the target populations, audience segmentation, tailored messaging, selection of appropriate channels, and commercial marketing approaches to ensure vaccines could be delivered via convenient and accessible services [13-16]. Program planning efforts would include continual analysis of the information landscape for competition, including misinformation and disinformation [15,17]. To build trust and engage young audiences often complacent about individual risk for disease, vaccine communication should be 2-way—listening and telling in equal measures—and in-person as well as web-based [13]. In urban communities, initiatives began by acknowledging historical injustices and ongoing inequities that drive distrust, with community-based health educators deployed to listen to concerns and provide support in person [18].

As a tool for providing tailored messaging, social listening, and 2-way dialogue, automated conversational agents, or chatbots, were cited early in the pandemic as a promising tool to offer COVID-19 health guidance on demand [19,20]. Chatbots have provided support on a range of health issues including chronic disease, addiction, reproductive health, depression, and anxiety, with promising adaptations of evidence-based interventions such as cognitive behavioral therapy [21-25]. This approach may appeal especially to young adults, since a substantial proportion of millennials, born from 1981-1996, are more trusting of web-based information and better equipped to use health technology than earlier generations [26,27]. Since the start of the pandemic, chatbots have been designed to provide COVID-19 health guidance in experimental settings [28], with some available globally via messaging platforms such as WhatsApp and Telegram [29,30]. Given their engaging, dialogue-based design, chatbots have a role in reinforcing public health guidance disseminated via other interventions such as social media campaigns. However, there is limited evidence related to the message design and framing of vaccine-related content delivered over digital platforms (eg, social media) and very little known about how such messaging should be delivered by educational chatbots in public health contexts [31].

Formative research has enabled the production of tailored content to optimize the delivery of messages [32]. An overarching factor in engaging and persuading audiences is the presence of credibility and trust, which can be defined as a combination of integrity, dependability, and competence [33,34]. Continually assessed by audiences, credibility can be lost through the delivery of a muddled or apparently dishonest message. Perhaps most central to vaccine communication in the context of hesitancy is the use of empathy, or a sense of one speaker understanding the experience of another. Empathic and reflective statements are critical components in motivational interviews, one of the few evidence-based means to soften vaccine hesitancy [35,36].

Seeking to review the acceptability of messages to be delivered by a chatbot, we engaged with potential users to identify which styles were preferred by young, urban-dwelling Americans. We also studied message reception with public health workers, anticipating that the chatbot would be used by the latter as a job aid. This formative research supported the development of a COVID-19 vaccine chatbot, VIRA, which was launched in 2021 by the International Vaccine Access Center at the Johns Hopkins Bloomberg School of Public Health [37]. IBM Research developed and managed the chatbot’s back end, which used Key Point Analysis, a commercially available technology that facilitates “extractive summarization” to process numerous comments, opinions, and statements and reveal the most important points and their relative prevalence. VIRA was initially programmed to respond to 100 Key Points, with up to 4 styles of responses to each identified concern. Key Points or distinct vaccine concerns were identified through various means: conducting a Twitter analysis, reviewing audience questions in Zoom-based public forums hosted by our affiliated academic centers, and synthesizing web pages with frequently asked questions [38-40]. To draft responses, we considered previous evidence that emphasizing social and physical consequences in an emotional format elicits broad influence [14], as well as evidence that trust is established through the perceptions of care and concern [41,42]. VIRA’s response database initially consisted of factual-only responses (responses containing data-driven information), empathy-factual responses (factual responses beginning with an empathetic phrase that validated the user’s query), principled responses (responses that appeal to a user’s conscience, often referencing community well-being [43]), rational arguments (responses containing a logical argument), testimonials (responses featuring a quote from a reputable expert), and humorous responses. All responses sought to minimize technical language and word count (under 280 characters). In this analysis, we investigated the appropriateness and tailoring of these responses.

## Methods

### Recruitment

Through 4 semistructured focus group discussions (FGDs), we assessed the appropriateness of different styles of responses to common COVID-19 vaccine questions. We selected focus groups to generate insightful participant discussions to illustrate group norms and diversity in the sampled population within a short period of time [44]. We recruited 2 participant groups in the United States: (1) urban-dwelling individuals aged 18-28 years and (2) public health workers, defined as individuals contracted or employed by health departments to encourage the uptake of COVID-19 vaccines. To identify health workers, we used snowball sampling through professional contacts in urban health departments of the United States. We also posted ads on Craigslist and Twitter, targeting both health workers and young people in Baltimore, Charlotte, New York City, Philadelphia, and Washington, D.C. We aimed to achieve maximum variability in race and ethnicity for both participant groups to explore attitudes toward chatbots providing health information. Since the chatbot was aimed to support people along the continuum of vaccine hesitancy up to vaccine refusal, and our study aimed to encourage productive group discussions among individuals with some openness to change around vaccination, we excluded people who stated they would “definitely NOT choose to get a COVID-19 vaccine by August 2021” in a scaled response [45,46].

### Ethics Approval

The Johns Hopkins Bloomberg School of Public Health Institutional Review Board approved this study (approval 15714).

### Data Collection

Following individually obtained informed consent, participants completed a demographic questionnaire using Qualtrics software (SAP America). Each participant then joined a web-based FGD via Zoom (licensed account; Zoom Video Communications Inc), 1 of which was composed exclusively of health workers and the other 3 composed of young participants [47]. Discussions were facilitated by a doctoral-level researcher, a masters-level faculty member, and a trained graduate student. Each FGD lasted 1 hour and had a maximum of 8 participants. Facilitators introduced VIRA, a chatbot developed by Johns Hopkins University that provided answers to common COVID-19 vaccine questions. Participants viewed 7 cards containing a question related to COVID-19 vaccination and 3 or 4 proposed responses written in various message styles. Participants were asked to select their preferred response, and conversation was encouraged between participants to further explore preferences. Table 1 displays sample messages (see Multimedia Appendix 1 for all message content).

**Table 1:** Sample questions and tested response styles.

Question or comment, response type		Response
**I’m not sure if the vaccine is safe, so I want to see how it affects others before I get vaccinated.**		
	Factual-only response	All vaccines go through clinical trials to test safety and effectiveness. For the COVID-19 vaccines, the FDA^a^ set up rigorous standards for vaccine developers to meet and thousands of people worldwide participated in clinical trials before the vaccines became available to the public!
	Empathy-factual response	This is an important question for many people! Once a vaccine is authorized for use, monitoring continues with systems in place to track problems or side-effects that were not detected during clinical trials. You can feel safe knowing these systems have got your back!
	Principled response	It’s very natural to have concerns. Yet, if some people choose to wait, we will not beat this pandemic any time soon. If you are willing to get vaccinated, you can do so knowing that millions have been safely vaccinated and you are helping our path to normalcy.
**I’m worried about vaccine side effects and adverse reactions.**		
	Rational argument	The likelihood of experiencing a severe side-effect is very small—less than 5 out of 1,000 people! You’ll probably just have some manageable side-effects that resolve in a few days.
**I’m young and healthy, so why do I need to get vaccinated?**		
	Testimonial	A Harvard physician said, “while the vast majority of young adults who get COVID-19 are not going to require hospitalization, those who do have a really high risk for adverse outcomes.” A vaccine can prevent severe illness, even if you’re young and healthy.
**Should I get the vaccine if I’ve had COVID-19?**		
	Humorous response	Spoiler: People who have COVID-19 should still get vaccinated, but only AFTER you get well!

^a^FDA: U.S. Food and Drug Administration.

### Data Analysis

Recorded FGDs were transcribed using Temi software [48], with investigators reviewing each transcript for quality assurance. Dedoose software (version 9.0.46; SocioCultural Research Consultants) [49] was used for data management, such as coding, code report exports, and the reassembly process. We used a grounded theory approach to develop a conceptual framework of how people respond to the various styles of COVID-19 vaccine messaging that could inform the subsequent production of chatbot responses [50].

Through our inductive qualitative analysis process, we identified the emerging themes with which to code our data [51]. First, we developed our initial codebook, with 2 researchers independently reviewing and conducting line-by-line coding of 2 rich transcripts and producing open codes. The study team then convened to review and condense these open codes into broader themes and subthemes. Once the codebook was finalized, 2 researchers then coded each transcript and a third resolved any coding discrepancies. Although some coding redundancy was discovered, no new codes were identified, indicating a saturation of the themes outlined within the codebook [52]. Once the coding process was complete, the team arrayed the data into matrices to identify thematic patterns related to the code “chatbot credibility”—discussions of which were woven throughout FGDs, as seen in memos produced during coding and reassembly. To understand participants’ overall preference for certain message styles, we also produced a count of preferences for each message type reviewed during the FGD process.

## Results

### Participant Characteristics

Between June and October 2021, several months after COVID-19 vaccines became widely available in the United States, we held 4 web-based focus groups with a total of 7 individuals aged 25-61 years working in public health or vaccine outreach roles and 13 people aged 18-28 years (see Table 2 for participant demographics). Of the 20 participants, most (80%, n=16) were women, with a mean age of 28.5 years. The median self-reported household income was US $56,000; for younger participants, this likely included parental income. Most (90%, n=18) participants said they were vaccinated against COVID-19.

**Table 2:** Participant demographics.

Characteristic		Participant
**Self-identified gender (N=20), n (%)**		
	Female	16 (80)
	Male	4 (20)
**Highest level of education (N=20), n (%)**		
	High school graduate	4 (20)
	Some college, no degree	3 (15)
	Bachelor’s degree or higher	13 (65)
**Self-identified race or ethnicity (N=20), n (%)**		
	American Indian or Alaska Native	1 (5)
	Asian or Pacific Islander	4 (20)
	Black or African American	5 (25)
	Hispanic or Latino	3 (15)
	White	4 (20)
	More than 1 race or ethnicity	3 (15)
**Age group (years; N=20), n (%)**		
	18-29	15 (75)
	30-49	3 (15)
	50-69	2 (10)
Self-reported annual household income (2021; US $; n=19), median (range)		56,000 (25,000-200,000)
Vaccinated (N=20), n (%)		18 (90)

### Thematic Findings

Analysis of FGDs identified themes describing the message preferences of young adults and public health workers in urban American communities. In the following quotes, we describe participants as either public health workers (“advocates”) or young people.

#### Credibility

The credibility of a chatbot message was the predominant theme influencing response selection. Both young participants and advocates said messages achieved credibility through (1) message directness and (2) the establishment of rapport between the user and chatbot through conversational syntax and empathetic, reflective statements. Of the 26 total responses, 6 (23%) consisted of an empathy-factual composite style, beginning with a user-centered, reflective message such as “it sounds like you have concerns” or “this is an important question for a lot of people.” Empathetic responses used casual, nontechnical language to answer questions using evidence in what participants said was a transparent, contextualized response. A young woman described the style as, “kind of sticking to the facts in a colloquial/conversational manner—doesn’t feel like I’m reading a newspaper or a research paper” (Participant 09).

Another young woman liked the conversational tone and “extensive” detail provided in a factual-style message about side effects, which stated that the vaccine’s side effects “should resolve within one or two days of vaccination.” She said, “It covered it pretty extensively. It sets me up for what I can expect. And then I would feel more secure knowing...the chatbot, it’s giving me [a] correct answer” (Participant 19).

**Figure 1:**
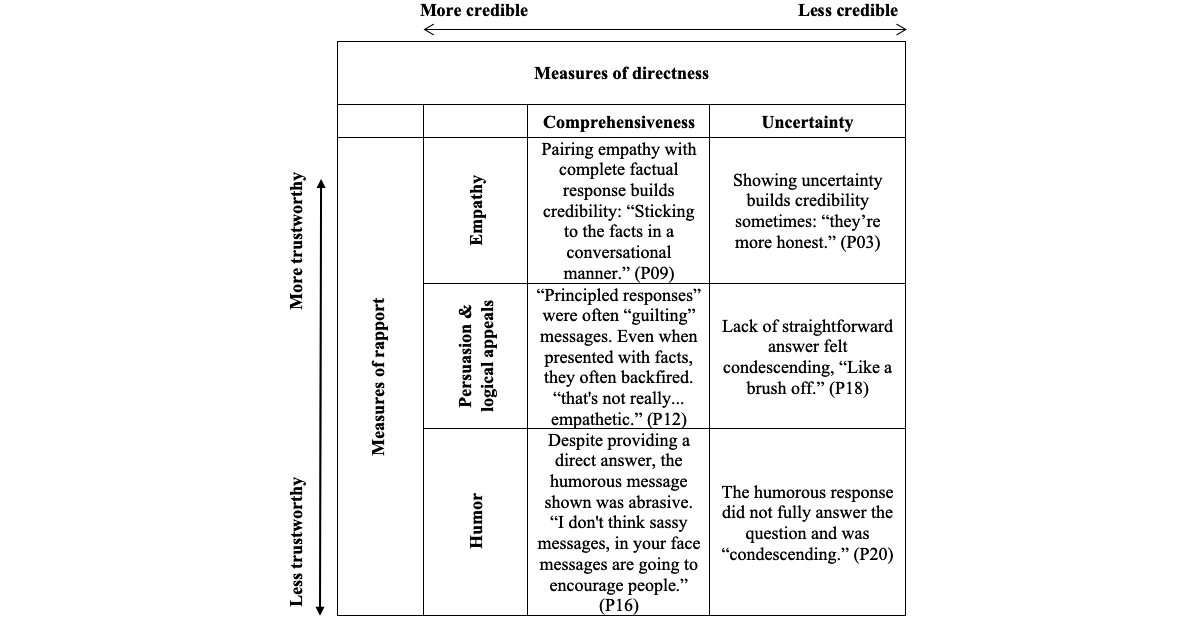
Message attributes supporting and hindering credibility with young focus group participants. Textual excerpts coded with both directness- and rapport-related variables (eg, each cell shows a textual passage double coded with a directness-related variable and rapport-related variable). P: participant.

Figure 1 illustrates how messages achieved credibility through rapport-building and directness and how participants felt messages lost credibility when responses didn’t answer a question directly—“like a brush off.” As seen in textual excerpts in the table, trustworthiness was eroded when messages compromised rapport, either by incorporating humor or by deploying guilt-laden arguments.

Both groups of participants regarded scientific messages as credible, saying they trusted the message since it was communicated by a Johns Hopkins University chatbot. In a typical response about how the brand affected message reception, a young man said: “I felt this [was] trustworthy, [be]cause I knew it came from like Johns Hopkins, which has a strong reputation” (Participant 07).

Although advocates and young participants both preferred empathetic statements prefacing a full, factual response, some felt such messages seemed inauthentic. The phrase “having doubts is normal,” in the words of a male advocate, “makes [the chatbot] more humane, more human-like, and more accepting” (Participant 08). Meanwhile, participants felt the phrase “I hear you” was marginally reassuring, but the statement “I wondered about that too” seemed “weird and fake [from a chatbot]” to one young woman (Participant 05).

#### Responsibility

Principled responses sought to persuade users to get vaccinated by appealing to an individual’s responsibility for the well-being of a larger community and using reasoning instead of evidence (see example in Table 1). For instance, instead of stating that vaccines are safe because “All vaccines go through clinical trials” as in a factual response, a principled response would say, “If some people chose to wait, we will not beat this pandemic any time soon.” Young participants saw this as evasive as well as “condescending” and “aggressive.” In the words of several participants, these messages were judgmental or shaming; as told by a young participant “You’re telling them that you put your families, your community at risk too. You’re making them feel guilty...I feel like that’s not really...empathetic” (Participant 12).

However, the style had some appeal with 4 young participants of color. One participant, a young man, felt such direct messaging was warranted:

I like [principled response] D because at this point in time, I think we need more aggressive messaging. Like, guilt people, shake people, let’s be serious...don’t put your friends and family at risk. Just get the shot.

Participant 10

Meanwhile, advocates aged >30 years often preferred such messages, sometimes wanting messaging to be “stronger” and “louder” to combat misinformation around vaccine myths. As one advocate said, “I think that message should be really pushed out a little louder. [The vaccine] prevents you from getting deathly ill” (Participant 16).

Advocates preferred principled responses that emphasized shared responsibility to prevent COVID-19 spread. As one young advocate said, these responses “made me think about the risks I posed not just [for] myself, but the people around me” (Participant 01). Moreover, advocates shared concerns about their family members and discussed feeling surrounded by people that “weren’t doing their part.” As one advocate said, “We’ve been getting hit hard, especially in the Black and Indigenous, Latinx, API communities, and this thing isn’t going anywhere anytime soon” (Participant 18).

An advocate recalled seeing community members previously hospitalized with COVID-19 “still not wearing your mask...it just made me more weary” (Participant 17). Such fatigue with community members not taking precautions to protect themselves and one another was linked with participant preference for principled messaging that was direct and insistent on communal responsibilities.

#### Resistance to Logical Appeals

Participants rarely preferred the rational arguments shown. We tested the following message in response to the question, “Are COVID vaccines worse than the disease itself?”

The trouble with that logic is that it’s difficult to predict who will survive an infection without becoming a COVID-19 long hauler. Almost 30% of people who’ve survived COVID-19 still experience long-term side-effects!

One participant commented that the tone of the response sounded “judgmental.” Similarly, a young woman said “it was like the most convincing argument as to why you would want to get the vaccine because like it shows how many people get long-term side effects, but I did agree that the tone...was a little condescending” (Participant 20).

#### Humor

Almost unanimously, participants disapproved of the humorous response shown and said it mocked people for asking questions. Both young people and advocates said it was “pushy,” “saucy,” and “condescending.” Moreover, participants said it did not fully answer the question or provide context to support statements.

#### Balancing Comprehensiveness and Uncertainty

After “comprehensiveness,” the code “credibility” was most likely to overlap with “transparency,” indicating the importance of directly answering a question without seeming to withhold information. Although both advocates and young people discussed wanting responses to be both direct and comprehensive, the participants did not agree about explicitly highlighted scientific uncertainty. For instance, one message said, “scientists aren’t totally sure” about whether vaccines stop transmission. Advocates said acknowledging ongoing studies was appropriate, since “we’re still learning about it,” in the words of an advocate (Participant 18). However, young participants disagreed, saying phrases acknowledging uncertainty were unsettling and did not promote vaccination, with a young participant stating “she [the chatbot] seemed super uncertain” or “it almost makes it seem like people should wait to see more studies [to get the vaccine]” (Participant 19).

#### Authority as Elitism

Young participants considered the use of a testimonial-style quote attributed to a Harvard physician to be elitist. One male advocate responded by saying, “Why do I care? It’s throw[ing] that he just has a title at my face” (Participant 08). Advocates aged >30 years agreed that the testimonial was not helpful, citing the politicization of doctors and science and suggesting the chatbot display testimonials from frontline health workers, such as emergency medical technicians.

### Relative Message Preference

To triangulate and strengthen our qualitative analysis, we tallied the number of votes the participants cast indicating their preferences for the messages on each of the 7 cards shown. FGD participants voted for a preferred message a total of 84 times (some did not select a response for each card shown). Participants voted for empathetic-factual messages 40 (48%) times, over 50% more times than factual-only messages—which at 24 (29%) votes was the second most preferred message style. However, although young participants most often (51%, 31/61) selected the empathetic-factual messages presented on message cards, public health workers most often (38%, 8/21) selected a principled response; young people rarely (8%, 5/61) selected this style with a few exceptions described above. Participants infrequently (8%, 7/84) preferred rational arguments, and participants never selected the testimonial or humorous messages, although just 1 example of each were shown on the cards. Although these quantitative results are not statistically significant given the qualitative study design and small sample size, the overwhelming preference for empathetic-framed responses among young participants is notable.

## Discussion

### Principal Findings

In this formative study of preferences for messages delivered by a COVID-19 chatbot, participants from urban American communities favored messages that were empathetic, direct, and comprehensive in answering questions related to COVID-19 vaccination. Messages achieved credibility through a combination of empathy and straightforward, evidence-based responses. User-centered reflective statements and conversational language that minimized the use of technical jargon fostered rapport between the chatbot and user.

Public health messages often contain statistics and rational statements, a strategy shown to effectively counter vaccine misinformation [53]. However, among our participants, most of whom were already vaccinated, this strategy alone was not as successful as messages that also included empathetic statements. Other studies among Black Americans with chronic conditions during COVID-19 found that participants also preferred chatbots to be “personable and empathetic” [54]. Other empathy-simulating chatbots have reported similar positive feedback from users [55-57]. Empathetic statements validated people’s search for knowledge and implicitly acknowledged the loneliness of the pandemic experience [58].

Participants in our study preferred straightforward, comprehensive responses that are similar to answers from an informed and respectful human interlocutor. The chatbot needed to completely answer user questions, or else may be perceived as evasive and potentially untrustworthy. Such expectations for politeness and respect align with the observations of technological anthropomorphizing, building on studies that show individuals’ interactions with computers are “fundamentally social” and that people naturally characterize the computer as a social actor [59-61].

For most young study participants, principled responses—messages appealing to concerns for family and community—counteracted the chatbot’s attempts to simulate empathy. In contrast, a meta-analysis of 60 studies found that younger audiences were the group most influenced by messages highlighting social consequences, showing the complexity of parsing message tactics in vaccine science when layered on top of a pandemic context [62]. However, for public health workers and several young people of color, the strong appeal to solidarity resonated with pandemic fatigue and frustration with individuals remaining unvaccinated in the face of widespread community distress; this finding is reinforced by the meta-analysis, which showed that so-called high-involvement audiences prefer data and strong messages that are somewhat fearful [62]. Built with audience-tested messages, the chatbot could offer a framework to support communication between health workers and community members that would integrate facilitated empathy.

Similarly, “rational arguments” eroded rapport between participants and the chatbot. Social media–related studies have used similar framing to “inoculate” audiences against misinformation, but this approach was not well-received in our study in the context of a chatbot [63,64].

The testimonial message shown to participants in the study was unappealing because the spokesperson was viewed as elitist. Future testimonials used in chatbot messages could be matched to participant demographics [65].

Although the humorous message shown to participants in this study performed poorly, humor is increasingly used to reach social media audiences otherwise disengaged from a public health topic and promote widespread sharing [66-68]. Friendly, self-deprecating humor as seen in popular voice assistant bots may be a better choice for one-to-one anonymous conversations with a chatbot than meme-style humor [69].

### Limitations

Although this study revealed COVID-19 vaccine message preferences for chatbot conversations with young, urban community members in the United States, it has several limitations. First, our participants were mostly college-educated. Given our reliance on Twitter ads for recruitment, this may be because Twitter users tend to be better educated and more left-leaning [70]. In addition, our chatbot’s institutional branding, widely known to promote pandemic mitigation measures, likely dissuaded some vaccine skeptics. Despite the fact that most participants were vaccinated, a range of concerns regarding COVID-19 vaccines was proffered, and we regard hesitancy as a reflection of a wide spectrum of concerns and views, including among those deciding to get vaccinated. The uncertainties of the pandemic were challenging to our study’s feasibility. Since we needed to collect data rapidly to iteratively redesign a tool already in use by public health departments, we held only 4 focus groups, limiting the potential transferability of the findings to similar populations in other geographic areas or among other communities. However, due to the observed permeability of the US population segments of vaccinated and unvaccinated people, we believe the results are relevant to support efforts to counteract vaccine hesitancy [6]. The pandemic was a highly dynamic environment for studying a tool to counter vaccine hesitancy, and the well-publicized, highly contagious delta variant spread was concurrent with recruitment, increasing the uptake of vaccines; in the subsequent months, residual concerns in vaccinated members of the public have surfaced as well as the reluctance to get booster doses [71]. Further, most participants identified with or worked in communities with high rates of vaccine hesitancy and referenced the concerns of community members.

Moreover, preferences may not predict web-based behavior, and the appraisal of messages in the FGDs may diverge from assessments in the context of a dialogue-based chatbot [72]; additionally, due to the constraints of the hour-long discussions, we were unable to show multiple varieties of humor- and testimonial-style messages that may have yielded different findings. Other determinants of message acceptance, including the influence of gain- or loss-framing on chatbot message preference, could be further explored, and ultimately, the chatbot’s impact on actual behavior should be evaluated [62,73].

### Conclusions

This study focused on the establishment of credible messaging from a chatbot to be used by young Americans and public health workers during the peak of the COVID-19 pandemic. We found that for young audiences, message credibility was optimized with empathetic statements and comprehensive, direct, and evidence-rich content. Pandemic-weary advocates and some young people from communities disproportionately affected by COVID-19 tended to prefer stronger, responsibility-focused messaging. Although credibility is essential to persuading users of a position, persuasion was not the target of this study. Additional controlled and randomized studies are needed to determine if a chatbot could persuade users to change their perception of the safety and effectiveness of vaccines and get vaccinated.

## Appendix

Multimedia Appendix 1 Full range of messages shown to focus group participants.

## Abbreviations

FGD: focus group discussion
