# Supplementary material for: Chatbot-Delivered COVID-19 Vaccine Communication Message Preferences of Young Adults and Public Health Workers in Urban American Communities: Qualitative Study
Source: J Med Internet Res. 2022 Jul 6;24(7):e38418. doi: 10.2196/38418 (PMC9301547; doi:10.2196/38418)
Supplement: Multimedia Appendix 1 [file jmir_v24i7e38418_app1.docx]

**Multimedia Appendix 1: Messages shown to focus group participants.**

**Question 1: Are the COVID-19 vaccines worse than the disease itself?**

1. **Factual**: A mountain of evidence shows that getting infected with the virus is far more dangerous than the vaccines. DYK almost 30% of people who’ve survived COVID-19 still experience long-term side effects?!​
2. **Factual/rational argument:** The trouble with that logic is that it’s difficult to predict who will survive an infection without becoming a COVID-19 long hauler. Almost 30% of people who’ve survived COVID-19 still experience long-term side effects!​
3. **Empathy/factual:** Having doubts is normal! Here’s some info for you: in addition to feeling sick or being hospitalized, a COVID-19 infection can lead to long-term effects like chronic fatigue, brain fog, blood clots, and heart, lung, and kidney damage. The vaccine is far safer and won’t cause any of these issues!​
4. **Principled:** When you take chances with COVID-19, you put your friends, your family, and your community at risk, too. Even if you don’t feel sick, those around you could experience severe illness, hospitalization, and even death. Don’t take the risk – get vaccinated.

**Question 2: I’m young and healthy, so why do I need to get vaccinated?​**

1. **Factual:** Although you are less likely to be hospitalized or die because of COVID-19, people in their 20s, 30s, and 40s can still catch the virus and develop severe and lasting symptoms. The risk of COVID-19 isn’t worth it.​
2. **Testimonial:** Scott Solomon, a Harvard physician, says, “while the vast majority of young adults who get COVID-19 are not going to require hospitalization, those who do have a really high risk for adverse outcomes.” A vaccine can prevent severe illness, even if you're young and healthy. ​
3. **Empathy/factual:** A lot of people ask me that. Here’s what you need to know: young adults have had some of the highest rates of COVID-19 during the pandemic, with many requiring hospitalization or suffering long-term side effects. A COVID-19 vaccine is the best way to prevent you from getting very sick.​
4. **Principled:** Many people in the US are still getting sick with COVID-19. Even if you, your family, or your friends haven’t experienced it firsthand, that doesn’t mean COVID-19 isn’t a threat. Getting vaccinated can protect you and our community against the COVID-19 pandemic.

**Question 3: I’m not sure if the vaccine is safe, so I want to see how it affects others before I get vaccinated.​**

1. **Factual:** All vaccines go through clinical trials to test safety and effectiveness. For the COVID-19 vaccines, the FDA set up rigorous standards for vaccine developers to meet, and thousands of people worldwide participated in clinical trials before the vaccines became available to the public!​
2. **Factual:** Over 100,000 people have taken part in COVID-19 vaccine trials, helping to speed up efforts to discover a safe and effective vaccine. Worldwide, millions have already been safely vaccinated and the vaccine has been shown to be safe.​
3. **Empathy/factual**: This is an important question for many people! Once a vaccine is authorized for use, monitoring continues with systems in place to track problems or side effects that were not detected during clinical trials. You can feel safe knowing these systems have got your back!​
4. **Empathy/Principled:** It’s very natural to have concerns. Yet, if some people choose to wait, we will not beat this pandemic any time soon. If you are willing to get vaccinated, you can do so knowing the millions have been safely vaccinated and you are helping our path to normalcy.

**Question 4: I’m worried about vaccine side effects and adverse reactions.​**

1. **Factual:** Like other vaccines, some people have temporary side effects like soreness, headache, fatigue, body aches, chills, and low-grade fever. These should resolve within one or two days of vaccination.​
2. **Factual**: Over 145 million people in the US have been vaccinated and it seems like side effects are pretty manageable and similar to other vaccines!​
3. **Empathy/factual**: It’s understandable to be worried about this. Short-term side effects like fever or soreness are normal signs that your body is building protection.​
4. **Rational argument**: The likelihood of experiencing a severe side-effect is very small – less than 5 out of 1,000 people! You’ll probably just have some manageable side effects that resolve in a few days.

**Question 5: Should I get the vaccine if I’ve had COVID-19?**

1. **Factual:** You may gain additional protection from the vaccine. Immunity from having had COVID-19 may not last very long, but more studies are needed to better understand this.​
2. **Empathy/rational argument:** I wondered about that, too. People who have recovered from COVID-19 should still consider a vaccine because we don’t know how long immunity from the virus lasts. Don’t take the risk!​
3. **Humor/rational argument:** Spoiler: People who have COVID-19 should still get vaccinated, but only AFTER you get well!​
4. **Principled:** Even if you had COVID-19, there’s still a chance that your immunity won’t last very long. In this case, COVID-19 will pose a risk to you and your loved ones, so you should ask your doctor about getting the vaccine.

**Question 6: Does the vaccine prevent me from giving the virus to others?**

1. **Factual:** Yep! Because the vaccine helps prevent you from getting infected, it also helps prevent you from spreading COVID-19 to others. ​
2. **Empathy/factual:** Great question – here's what I found! COVID-19 vaccines are effective at keeping you from getting sick and reducing symptoms even if you get infected. Scientists are still studying how well vaccines prevent spreading of the virus to others, but evidence suggests vaccines prevent transmission!​
3. **Principled:** Researchers aren’t totally sure. Which is why, even though COVID-19 vaccines are effective at keeping you from getting sick, we need to maintain safety measures (like wearing a mask) in crowded places. This will keep unvaccinated members of your community safe.

**Question 7: What is in the vaccine?​**

1. **Factual**: Some of the vaccines use mRNA. Researchers have studied mRNA vaccines for decades. The mRNA in the vaccine lasts up to 24 hours in the body and then is broken down. Viral vector vaccines— AstraZeneca or Johnson & Johnson vaccines—use a safe virus to deliver the instructions to your body.​
2. **Empathy/factual**: It sounds like you have concerns. It’s normal to wonder about this! The most important ingredient in both the Pfizer and Moderna vaccines is mRNA. The mRNA tells your cells how to recognize COVID-19 and fight it off.​
3. **Factual**: COVID-19 vaccines don't include any toxic ingredients. The leading vaccines are made of mRNA, lipids (fats that encase and protect the mRNA), salts, and sugar.
